# Supplementary material for: Strong interlayer coupling and stable topological flat bands in twisted bilayer photonic Moiré superlattices
Source: Light Sci Appl. 2022 Oct 6;11:289. doi: 10.1038/s41377-022-00977-4 (PMC9537166; doi:10.1038/s41377-022-00977-4)
Supplement: Supplementary file 1 — Supplementary Information for “Strong Interlayer Coupling and Stable Topological Flat Bands in Twisted Bilayer Photonic Moire ´ Superlattices” [file 41377_2022_977_MOESM1_ESM.pdf]

Supplementary Information for
"Strong Interlayer Coupling and Stable Topological Flat Bands in Twisted Bilayer Photonic Moiré Superlattices"

Chang-Hwan Yi,* Hee Chul Park,[†a] Moon Jip Park[†b]

Center for Theoretical Physics of Complex Systems, Institute for Basic Science (IBS),
Daejeon, 34126, Republic of Korea
E-mail: *yichanghwan@hanmail.net, [†a]hcpark@ibs.re.kr, [†b]moonjippark@ibs.re.kr
[†]Corresponding authors equally contributing to this work.

Contents

Page

S1 Construction of the effective tight-binding model 3

S2 Symmetry class of the Helmholtz equation 4

S3 Quantization of the polarization in the presence of the reflection symmetry 5

S4 Equivalence of the reflection protected polarization and the second Stiefel-Whitney number 7

S5 Band representations based on the topological quantum chemistry 7

S6 Detailed calculation of the photonic band structure 11

S7 **Boundary element method** **11**

S8 **Block Sakurai–Sugiura contour integral method** **15**

S9 **Periodic boundary conditions for 1D and 2D structures** **16**

S10 **Boundary shape of a single resonator quasi-atom** **17**

S11 **Full characterization of the wave functions and parity eigenvalues at the TRIM points** **19**

S12 **Bulk, edge, and corner modes in a finite size system under a “dielectric” boundary condition** **23**

S13 **Edge and corner modes under the “Dirichlet” boundary condition** **24**

S14 **Energy bands depending on the refractive index of resonator quasi-atoms** **26**

S15 **Hopping strength depending on the refractive index** **27**

S16 **Twist angle-dependent energy bands** **28**

S17 **Twist angle-dependent density of states (DOS)** **29**

S18 **Tunable intra and interlayer couplings as a function of the twist angle** **30**

S19 **Strongly localized waves on the innermost dodecagonal quasi-atoms associated with flat bands** **31**

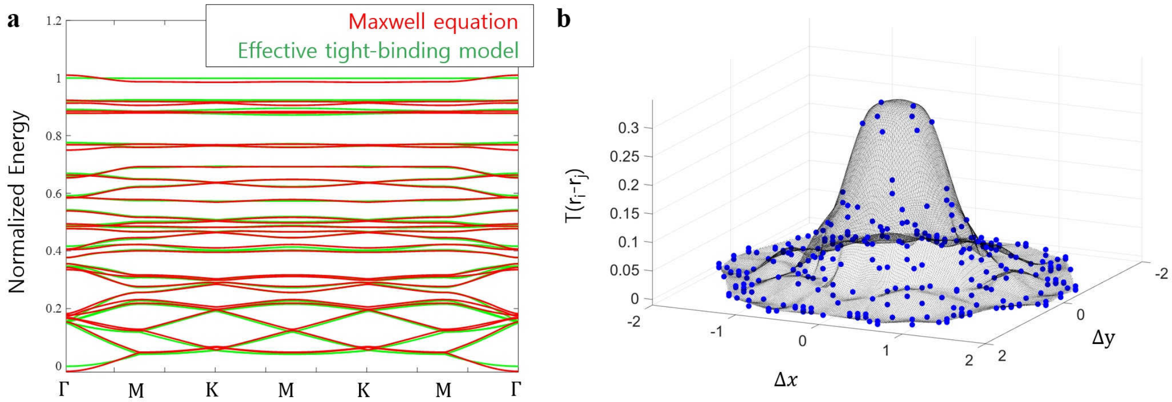

Figure S1: **a** Comparison of the band structures derived from the Maxwell equation (red) and the effective tight-binding model (green). **b** Effective hopping strength as a function of the displacement between two quasi-atoms.

S1 Construction of the effective tight-binding model

To construct the effective tight-binding model, we extract the band energy and the wave functions at the time-reversal invariant momenta ($\mathbf{k}_{\mathrm{TRIM}} \in \{\Gamma, \mathrm{M}^{+}, \mathrm{M}^{-}, \mathrm{M}^{0}\}$) from the solution of the Helmholtz equation. Then, the Bloch Hamiltonian of the lowest 28 bands can be reconstructed as

$$H(\mathbf{k}_{\mathrm{TRIM}}) = U(\mathbf{k}_{\mathrm{TRIM}})D(\mathbf{k}_{\mathrm{TRIM}})U^{\dagger}(\mathbf{k}_{\mathrm{TRIM}}), \tag{S1}$$

where $D(\mathbf{k})$ is the diagonal matrix consisting of the energies of the lowest 28 bands, and $U(\mathbf{k}_{\mathrm{TRIM}})$ is the unitary matrix containing the wave functions. More specifically, $U(\mathbf{k}_{\mathrm{TRIM}})$ is 28×28 where the element U_{ij} is given as

$$U_{ij} = \int_i dA \Psi_j, \tag{S2}$$

where Ψ_j is the solution of the Helmholtz equation for the j-th band. Area integration is performed in the interior region of the i-th resonator.

After deriving the Bloch Hamiltonian at the TRIM points, we can reconstruct the real space

tight-binding model. To do so, we notice that the Bloch Hamiltonian can be expressed by systems of the linear equations for the hopping matrices as follows:

$$\begin{aligned} H(\Gamma) &= H_{(0,0)} + H_{(\pm1,0)} + H_{(0,\pm1)} + H_{(\pm1,\pm1)} , \\ H(\mathbf{M}^+) &= H_{(0,0)} - H_{(\pm1,0)} + H_{(0,\pm1)} - H_{(\pm1,\pm1)} , \\ H(\mathbf{M}^-) &= H_{(0,0)} + H_{(\pm1,0)} - H_{(0,\pm1)} - H_{(\pm1,\pm1)} , \\ H(\mathbf{M}^0) &= H_{(0,0)} - H_{(\pm1,0)} - H_{(0,\pm1)} + H_{(\pm1,\pm1)} , \end{aligned} \tag{S3}$$

where $H_{(m,n)}$ is the hopping matrix from a moiré unit cell to the neighbor unit cell translated by the vector $\vec{L} = m\mathbf{b}_1 + n\mathbf{b}_2$. Hence, $H_{(0,0)}$ is the hopping matrix within the same unit cell. In addition to the above equations, the translational symmetry imposes the condition $H_{(m,n)} = H^\dagger_{(n,m)}$. With all these conditions, we are able to solve the above systems of the linear equations to derive the hopping strength between different sites. Figure S1 shows a comparison of the energy bands derived from the full Helmholtz equations and from the effective tight-binding model. We find the two results to agree quite well.

S2 Symmetry class of the Helmholtz equation

For a time harmonic field $\vec{\Psi}(\vec{r},t) = \vec{\psi}(\vec{r})e^{i\omega t}$, both the magnetic and electric fields can be expressed by the Maxwell equations, which are written respectively as,

$$\nabla \times \left[\frac{1}{n^2(\mathbf{r})}\nabla \times \mathbf{H}(\mathbf{r})\right] = \left(\frac{\omega}{c}\right)^2 \mathbf{H}(\mathbf{r}), \tag{S4}$$

$$\nabla \times \left[\frac{1}{n^2(\mathbf{r})}\nabla \times \mathbf{E}(\mathbf{r})\right] = \left(\frac{\omega}{c}\right)^2 \mathbf{E}(\mathbf{r}), \tag{S5}$$

where $n(\mathbf{r})$ is the spatially varying refractive index. Due to the absence of the divergence of the magnetic field [$\nabla \cdot \mathbf{H}(\mathbf{r}) = 0$] and the absence of the net electric charge [$\nabla \cdot \mathbf{E}(\mathbf{r}) = 0$], both

equations are described by the equivalent Helmholtz equation which is given as,

$$-\nabla^2\vec{\psi}(\mathbf{r}) = n^2(\mathbf{r})\frac{\omega^2}{c^2}\vec{\psi}(\mathbf{r})\ , \tag{S6}$$

where $\vec{\psi}(\mathbf{r}) \in \{\mathbf{E}(\mathbf{r}), \mathbf{H}(\mathbf{r})\}$. Now, for two-dimensional systems (or equivalently infinite-long cylinders), the TM and TE modes can be decoupled so that we can obtain them separately by considering $\mathbf{E} = (0, 0, E_z)$ and $\mathbf{H} = (0, 0, H_z)$, where E_z and H_z are the electric and magnetic fields that polarized along z-direction. Without loss of generality, both of the equations can be viewed as the eigenvalue equation, $\hat{H}\psi(\mathbf{r}) = E\psi(\mathbf{r})$ for $E = (\frac{\omega}{c})^2$, where $\hat{H} \equiv \nabla\times\frac{1}{n(\mathbf{r})^2}\nabla\times$ is the differential operator. Since the TM and TE modes that are governed by the two Helmholtz equations in Eqs. S4 and S5 are decoupled, each of them independently preserves the effective spinless time-reversal symmetry, $\mathcal{T} = \mathcal{K}$ as,

$$\mathcal{T}\hat{H}\mathcal{T}^{-1} = \hat{H}^* = \hat{H} \tag{S7}$$

where $\mathcal{K}$ is the complex conjugation operator. According to the Altland-Zirnbauer classification, the eigenvalue equation of $\hat{H}$ belongs to AI class since $\mathcal{T}^2 = 1$, which matches with the symmetry classifications of the spinless electronic systems (e.g., graphene).

S3 Quantization of the polarization in the presence of the reflection symmetry

In this section, we show the quantization of the polarization in the presence of the reflection symmetry. To start our discussion, we explicitly restate the expression of Eq. (4) in the main text. According to Resta's apporach, the polarization in each symmetry sector in the periodic lattice is formally defined as,

$$\nu_\pm = i\oint_{\mathbf{k}\in\Gamma-M-\Gamma} d\mathbf{k}\mathcal{A}(k_x, k_y)_\pm, \tag{S8}$$

where cyclic integration is performed along the reflection symmetric line, $\Gamma - M^0 - \Gamma$. $\mathcal{A}(k_x, k_y)_{\pm} = \langle \Psi_{\pm}(k_x, k_y)|\partial_k|\Psi_{\pm}(k_x, k_y)\rangle$ is the Berry connection defined for each reflection sectors of $\mathcal{M}_x$. $\Psi_{\pm}(k_x, k_y)$ is the Bloch wave function defined in the momentum space, which belong to the each symmetry sectors. In a finite system with discrete momentum vectors, we can equivalently write down the Berry connection as the innerproducts of the states along the adiabatic transform as,

$$e^{i\nu_{\pm}} = \det \langle \Psi_{\pm,i}(\Gamma)|\,\mathcal{U}_{\Gamma - M^0 - \Gamma}\,|\Psi_{\pm,j}(\Gamma)\rangle, \tag{S9}$$

where

$$\mathcal{U}_{\overline{k_1 k_2}} = \sum_{a,b,c,..} |\Psi_{\pm,a,\mathbf{k}'_1}\rangle\langle\Psi_{\pm,a,\mathbf{k}'_1}|\Psi_{\pm,b,\mathbf{k}'_2}\rangle\langle\Psi_{\pm,b,\mathbf{k}'_2}| \ldots |\Psi_{\pm,c,\mathbf{k}'_3}\rangle\langle\Psi_{\pm,c,\mathbf{k}'_3}|. \tag{S10}$$

The subscript $\Psi_{\pm,i,\mathbf{k}}$ indicates i-th eigenstates in each sector at the momentum $\mathbf{k}$. $\mathbf{k_1}', \mathbf{k_2}', \ldots$ form a line $\overline{k_1 k_2}$ in BZ. Using the property, $\mathcal{M}_y^2 = 1$, we can re-write Eq. (S10) as,

$$\begin{aligned} \mathcal{U}_{\Gamma - M^0 - \Gamma} &= \mathcal{U}_{\overline{\Gamma M^0}}\mathcal{U}_{\overline{M^0 \Gamma'}} \\ &= \mathcal{U}_{\overline{\Gamma M^0}}\mathcal{M}_y(\mathcal{M}_y \mathcal{U}_{\overline{M^0 \Gamma'}}\mathcal{M}_y)\mathcal{M}_y \\ &= \mathcal{U}_{\overline{\Gamma M^0}}\mathcal{M}_y \mathcal{U}_{\overline{M^0 \Gamma}}\mathcal{M}_y, \end{aligned} \tag{S11}$$

Here, the notation $\Gamma' \equiv \Gamma + \mathbf{b_1} + \mathbf{b_2}$ is used to indicate Γ point in the second BZ to avoid the confusion between $\overline{\Gamma M^0}$ and $\overline{M^0 \Gamma'}$ lines. Using the above relation, the overall polarization can be re-expressed as,

$$e^{i\nu_{\pm}} = \sum_{a,b,c} \det \left[\langle\Psi_{\pm,i,\Gamma}|\mathcal{U}_{\overline{\Gamma M^0}}|\Psi_{\pm,a,M^0}\rangle\langle\Psi_{\pm,a,M^0}|\mathcal{M}_y|\Psi_{\pm,b,M^0}\rangle\langle\Psi_{\pm,b,M^0}|\mathcal{U}_{\overline{M^0 \Gamma}}|\Psi_{\pm,c,\Gamma}\rangle\langle\Psi_{\pm,c,\Gamma}|\mathcal{M}_y|\Psi_{\pm,j,\Gamma}\rangle\right] \tag{S12}$$

Using the commutation property of the determinant, we can cancel out $\mathcal{U}_{\overline{\Gamma M}}$ and $\mathcal{U}_{\overline{M \Gamma}}$ as,

$$\begin{aligned} e^{i\nu_{\pm}} &= \sum_a \det \left[\langle\Psi_{\pm,i,M^0}|\mathcal{M}_y|\Psi_{\pm,a,M^0}\rangle\langle\Psi_{\pm,a,\Gamma}|\mathcal{M}_y|\Psi_{\pm,j,\Gamma}\rangle\right] \\ &= \prod_i \zeta_i(M^0)\zeta_i(\Gamma) \end{aligned} \tag{S13}$$

where $\zeta_i(\mathbf{k})$ is the i-th eigenvalue of $\mathcal{M}_y$ operator at $\mathbf{k}$. Since $\mathcal{M}_y^2 = 1$, each ζ_i can only take a value of ± 1. As a result, $\nu_\pm$ can only take a value of either 0 or π, which gives rise to $\mathbb{Z}_2$ classification of the bulk polarizations (*1*). We note that the $\mathbb{Z}_2$ classification is rather general consequence of PT symmetric system in AI class under Altland-Zirnbauer classification (*2*).

S4 Equivalence of the reflection protected polarization and the second Stiefel-Whitney number

In the coexistence of the four symmetries, $\mathcal{M}_x, \mathcal{M}_y$, and $\mathcal{P} = \mathcal{M}_x \times \mathcal{M}_y$, we can formally equate the reflection protected polarization($\nu_{+(-)}$) and the second Stiefel-Whitney number ν_2 defined in Eq. (6) in the main text. Due to the mirror reflection symmetry we have, $\zeta_i(M^+) = \zeta_i(M^-)$. Combining with Eq. S13, we derive the expression of the polarization in terms of the inversion eigenvalues at TRIM points as,

$$e^{i\nu_\pm} = \prod_{i \in \mathcal{M}_x = \pm 1} \zeta_i(\Gamma)\zeta_i(M_1)\zeta_i(M_2)\zeta_i(M_3) \tag{S14}$$

Since $\nu_+ + \nu_- = 0 \, (\mathrm{mod}\, 2\pi)$, we formally relate the expression of the polarization to the second Stiefel-Whitney number as,

$$e^{i\nu_\pm} = \prod_{\Gamma_i \in \mathrm{TRIM}} (-1)^{[N^-(\Gamma_i)/2]} = (-1)^{\nu_2}. \tag{S15}$$

S5 Band representations based on the topological quantum chemistry

Figure S2(a) illustrates the quasi-atomic sites of the photonic crystal at the twist angle $\theta = 21.78°$. At the center of the unit cell, the system preserves two mirror-reflection symmetries $\mathcal{M}_x$ and $\mathcal{M}_y$ and their product $C_{2z} = \mathcal{M}_x \times \mathcal{M}_y$ (point group C_{2v}). The symmetry operations of the wavefunctions are given as,

$$\mathcal{M}_x \quad : \quad \psi(x, y) \rightarrow \psi(x, -y)\ , \tag{S16}$$

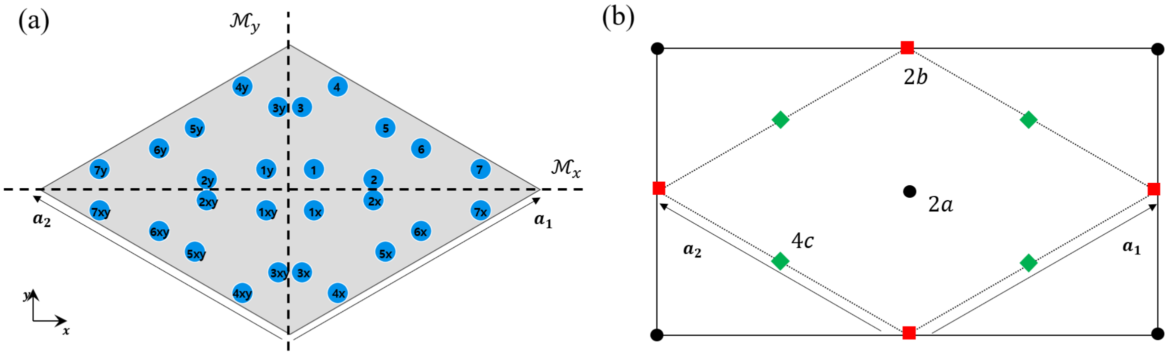

Figure S2: (a) Primitive unit cell of the twist angle $\theta = 21.78°$. There exist total 28 quasi-atoms. (b) Wyckoff positions of the unit cell. The maximal Wyckoff positions, 2a, 2b, 4c are indicated by black dots, red square, and green diamonds respectively.

$$\mathcal{M}_y \quad : \quad \psi(x,y) \to \psi(-x,y)\ ,$$

$$\mathcal{C}_{2z} \quad : \quad \psi(x,y) \to \psi(-x,-y)\ .$$

We note that the above symmetry operations hold for all commensurate twist angles. In addition, unlike the case of the AA-stacked twisted bilayer graphene, C_{3z}-symmetry is absent, since the overlapping sites (site number $2, 2_x, 2_y, 2_{xy}$) are slightly shifted toward y-direction in the M_{2x} and M_{2y} symmetric manner to avoid the overlap. The symmetry group of this photonic crystal is the three-dimensional space group $Cmm2$ (No. 35), generated by the lattice translation and C_{2v} point group. (2D symmetry group is recovered by taking z-directional length to infinity). Figure S2(b) illustrates the maximal Wyckoff positions of the space group $Cmm2$ (No. 35), where z-direction coordinate is ignored. There are three maximal Wyckoff positions: 2a, 2b, 4c.

To access the topological quantum chemistry, the symmetry transformation of Bloch eigenstates is examined at high symmetry points ($k_\star \in \{\Gamma, \mathrm{M}^{0,\pm}\}$), and we obtain the band structure labeled with the corresponding irreducible representations (irreps) of the little co-group in the high symmetry point $k_\star$ (see Tables S1 and S2 for the character table). Table S3 summarizes the symmetry operations and the corresponding irreps. Equipped with the full symmetry operations

and the irreps of all 28 bands, we now identify the band representations (BRs) of the interconnected energy bands separated by the global gap. There exist six global gaps separating seven interconnected band representations. We label each group of interconnected bands by $\mathcal{B}_\alpha$ where $\alpha = 1, .., 7$ (bands No. 1-10,11-17,18-21,22-23,24-25,26-27,28 respectively). The topological quantum chemistry can describe the following group of bands by identifying the symmetry transformation of the bands and the equivalent (composite) BRs, composed of the elementary band representations (EBRs). Table S4 shows EBR , derived from Bilbao Crystallographic Server (*3–5*).

By matching the symmetry operations of the total 28 bands with EBR listed in Table S4, we identify that $\mathcal{B}_{1,2,3,7}$ are the obstructed atomic limit (OAL) with the Wyckoff position 2b, while $\mathcal{B}_{4,5,6}$ are trivial. The explicit BRs consistent with the irreps of the 28 energy bands are given as,

$$
\begin{aligned}
\mathcal{B}_1 \quad &: \quad (\mathrm{A} \uparrow G)_{\mathrm{2c}} \oplus 4 \times (\mathrm{A}_1 \uparrow G)_{\mathrm{2a}} \oplus 2 \times (\mathrm{B}_1 \uparrow G)_{\mathrm{2a}} \oplus (\mathrm{B}_2 \uparrow G)_{\mathrm{2a}} \oplus (\mathrm{A}_2 \uparrow G)_{\mathrm{2b}} \\
&\rightarrow \quad \mathrm{OAL} \\
\mathcal{B}_2 \quad &: \quad (\mathrm{B} \uparrow G)_{\mathrm{2c}} \oplus (\mathrm{B} \uparrow G)_{\mathrm{2c}} \oplus (\mathrm{B}_2 \uparrow G)_{\mathrm{2a}} \oplus (\mathrm{B}_2 \uparrow G)_{\mathrm{2a}} \oplus (\mathrm{B}_1 \uparrow G)_{\mathrm{2b}} \\
&\rightarrow \quad \mathrm{OAL} \\
\mathcal{B}_3 \quad &: \quad (\mathrm{A} \uparrow G)_{\mathrm{2c}} \oplus (\mathrm{A}_2 \uparrow G)_{\mathrm{2a}} \oplus (\mathrm{B}_1 \uparrow G)_{\mathrm{2b}} \rightarrow \mathrm{OAL} \\
\mathcal{B}_4 \quad &: \quad (\mathrm{A}_1 \uparrow G)_{\mathrm{2b}} \oplus (\mathrm{A}_2 \uparrow G)_{\mathrm{2b}} \rightarrow \mathrm{Trivial} \\
\mathcal{B}_5 \quad &: \quad (\mathrm{A}_2 \uparrow G)_{\mathrm{2a}} \oplus (\mathrm{B}_2 \uparrow G)_{\mathrm{2a}} \rightarrow \mathrm{Trivial} \\
\mathcal{B}_6 \quad &: \quad (\mathrm{B}_1 \uparrow G)_{\mathrm{2b}} \oplus (\mathrm{B}_2 \uparrow G)_{\mathrm{2b}} \rightarrow \mathrm{Trivial} \\
\mathcal{B}_7 \quad &: \quad (\mathrm{A}_2 \uparrow G)_{\mathrm{2b}} \rightarrow \mathrm{OAL}
\end{aligned}
\tag{S17}
$$

In addition, we now show that the obstructed atomic limit (OAL) with the Wyckoff position

Rep	E	C_{2z}	$\mathcal{M}_x$	$\mathcal{M}_y$
A_1	1	1	1	1
A_2	1	1	-1	-1
B_1	1	-1	1	-1
B_2	1	-1	-1	1

Table S1: The character table for the little co-group (point group of the little group) $\bar{G}^{\Gamma,M^0} \approx C_{2v}$ for the TRIM point Γ and M^0.

Rep	E	C_{2z}
A	1	1
B	1	-1

Table S2: The character table for the little co-group $\bar{G}^{M^+} \approx C_2$ for the TRIM point $M^{\pm}$.

2b is equivalent to the non-trivial second Stiefel-Whitney insulator, discussed in Sec. S4.

Proposition 1 : The group of the interconnected energy band, $\mathcal{B}_\alpha$, transforms as BR of OAL with the Wyckoff position 2b if and only if the second Stiefel-Whitney (SW) number is non-trivial ($\nu_2 = 1$). The $\mathbb{Z}_2$-valued second SW number, ν_2, is defined as,

$$(-1)^{\nu_2} = \prod_{\Gamma_i \in \mathrm{TRIM}} (-1)^{[N_\alpha^-(\Gamma_i)/2]}, \tag{S18}$$

where $N_\alpha^-(\Gamma_i)$ is the number of bands in $\mathcal{B}_\alpha$ with $C_{2z} = -1$ at the TRIM Γ_i.

Proof : Among the irreps which belong to $G \approx C_2, C_{2v}$, B-class irreps (B, B_1, B_2) are odd under C_{2z} operations, while the remaining A-class irreps (A, A_1, A_2) are even under the C_{2z} operations. For each EBR, we count the total number of the irreps at TRIM points, which belongs the B-class irreps, n_B. By explicitly reading EBR in Table S4, EBRs with WP 2b have n_B=2, while EBRs with the other WPs has $n_B = 0$ or 4. Therefore, (composite) BR of OAL with WP 2b has $n_B = 4n + 2$ where n is an integer. Since n_B counts the number of the bands at the TRIM points with $C_{2z} = -1$, we find that $\sum_{\Gamma_i \in \mathrm{TRIM}} N_\alpha^-(\Gamma_i) = 4n + 2$, which corresponds to the non-trivial second SW number $\nu_2 = 1$.

$\Longrightarrow$ By combining **Proposition 1** and Sec. S4, we conclude that the following three phases

in the space group No. 35 are equivalent:

$$(1) \quad \text{Non-trivial second Stiefel-Whitney (SW) insulator.} \tag{S19}$$

(2) Non-trivial reflection protected polarization.

(3) Obstructed atomic limit (OAL) with the Wyckoff position 2b.

S6 Detailed calculation of the photonic band structure

To numerically compute the band structures of our two-dimensional photonic crystal, we utilize the so-called boundary element method (BEM) (*6, 7*), a discretized version of the boundary integral equation (BIE). The BEM is a powerful tool (*8*) for obtaining eigenstates (or quasi-normal modes or resonances) in piecewise homogeneous systems. This method does not require a full spatial domain mesh to calculate fields; it only needs to know the wavefunctions and their outward normal derivative on the domain boundaries of the involved subsystems. As the current state of BEM development is already in the textbook stage, we only briefly introduce a few of its core ideas here.

S7 Boundary element method

We can combine the free-space Green function G and unknown solution ψ given as solutions of the Helmholtz equations as

$$\left[\nabla^2 + n^2\frac{\omega^2}{c^2}\right] G\left(r, r'; \frac{\omega}{c}\right) = \delta(r - r') \ , \quad \left[\nabla^2 + n^2\frac{\omega^2}{c^2}\right] \psi\left(r; \frac{\omega}{c}\right) = 0 \tag{S20}$$

for a domain enclosed by a simple-connected-closed envelope Γ. Here, (r, r') is inside Γ, $G(r, r'; \omega/c) = -i/4H_0^{(1)}(n\omega|r-r'|/c)$ in two dimensions, and $\delta(r-r')$ is the two-dimensional Dirac delta function, where $H_0^{(1)}$ is the first kind Hankel function of order 0. The two equations

Table S3: Classification of the symmetry operations and the irreducible representations of each band for C_{2z} and $\mathcal{M}_{x,y}$ symmetry in the high symmetry TRIM point $(\Gamma, \mathrm{M}^0, \mathrm{M}^+, \mathrm{M}^-)$ of the Brillouin zone. Table output +(-) represents the even (odd) under the symmetry operation. The band separated by the single horizontal lines have distinct energy, while the bands not separated by the horizontal lines are degenerate. The bands separated by double horizontal lines have a global bandgap. The little co-group of Γ, M^0 ($\mathrm{M}^{\pm}$) corresponds to C_{2v} (C_2).

Band	Rep(C_{2v})	C_{2v}			Rep(M^0)	M^0			Rep(C_2)	M^+	M^-
		C_{2z}^{Γ}	$\mathcal{M}_x^{\Gamma}$	$\mathcal{M}_y^{\Gamma}$		$C_{2z}^{M^0}$	$\mathcal{M}_x^{M^0}$	$\mathcal{M}_y^{M^0}$		C_{2z}^{M+}	C_{2z}^{M-}
1	A_1	+	+	+	A_1	+	+	+	B	-	-
2	A_1	+	+	+	B_1	-	+	-	A	+	+
3	B_2	-	-	+	B_2	-	-	+	B	-	-
4	B_1	-	+	-	A_1	+	+	+	A	+	+
5	A_2	+	-	-	B_1	-	+	-	A	+	+
6	B_1	-	+	-	B_2	-	-	+	A	+	+
7	A_1	+	+	+	A_1	+	+	+	B	-	-
8	A_1	+	+	+	A_2	+	-	-	B	-	-
9	A_2	+	-	-	B_1	-	+	-	A	+	+
10	A_1	+	+	+	A_1	+	+	+	B	-	-
11	B_2	-	-	+	B_2	-	-	+	A	+	+
12	B_2	-	-	+	A_2	+	-	-	B	-	-
13	B_1	-	+	-	B_1	-	+	-	A	+	+
14	B_1	-	+	-	A_1	+	+	+	B	-	-
15	B_2	-	-	+	A_1	+	+	+	B	-	-
16	B_1	-	+	-	B_2	-	-	+	A	+	+
17	B_2	-	-	+	A_2	+	-	-	B	-	-
18	A_2	+	-	-	B_1	-	+	-	A	+	+
19	A_1	+	+	+	A_2	+	-	-	B	-	-
20	B_1	-	+	-	B_2	-	-	+	A	+	+
21	A_2	+	-	-	B_1	-	+	-	A	+	+
22	A_1	+	+	+	A_1	+	+	+	B	-	-
23	A_2	+	-	-	A_2	+	-	-	B	-	-
24	A_2	+	-	-	B_2	-	-	+	B	-	-
25	B_2	-	-	+	A_2	+	-	-	A	+	+
26	B_2	-	-	+	B_1	-	+	-	A	+	+
27	B_1	-	+	-	B_2	-	-	+	A	+	+
28	A_2	+	-	-	A_2	+	-	-	B	-	-

Table S4: Elementary band representations of the space group (No. 35). The first row represents the Wyckoff position (WP). The second row represents the band representations (BR). Since we consider the two-dimensional system, we only list down $k_z = 0$ momenta and ignore z-direction coordinates. Band representation output $G_\rho(n)$ indicates the induced representation $\rho \uparrow G(n)$ with the dimension n. The table is extracted from Bilbao Crystallographic Server (*3–5*)

WP	2a (0,0)				2b $(0\frac{1}{2})$				4c $(\frac{1}{4}\frac{1}{4}), (\frac{3}{4}\frac{1}{4})$	
BR	$G_{A_1}(1)$	$G_{A_2}(1)$	$G_{B_1}(1)$	$G_{B_2}(1)$	$G_{A_1}(1)$	$G_{A_2}(1)$	$G_{B_1}(1)$	$G_{B_2}(1)$	$G_A(2)$	$G_B(2)$
Γ	A_1	A_2	B_1	B_2	A_1	A_2	B_1	B_2	$A_1 \oplus A_2$	$B_2 \oplus B_1$
M^0	A_1	A_2	B_1	B_2	A_1	A_2	B_1	B_2	$B_2 \oplus B_1$	$A_2 \oplus A_1$
M^+	A	A	B	B	B	B	A	A	$A \oplus B$	$A \oplus B$
M^-	A	A	B	B	B	B	A	A	$A \oplus B$	$A \oplus B$

can be nested by subtracting one another after properly multiplying ψ and G to the two equations, as follows:

$$\psi\delta(r - r') = \psi\nabla^2 G - G\nabla^2\psi = \nabla\left[\psi\nabla G - G\nabla\psi\right]. \tag{S21}$$

The two-dimensional 'area integration' inside Γ gives ψ on the LHS, while we can reduce this domain integral to the 'line integraion' along Γ on the RHS by applying Green's second identity. Then, it explicitly induces a boundary integral equation for unknown $\psi(s)$, $\partial_\nu\psi(s)$, and ω/c, as follows:

$$\alpha\psi(r') = \oint_\Gamma ds\left[-G\left(s, r'; \frac{\omega}{c}\right)\partial_\nu\psi(s) + \psi(s)\partial_\nu G\left(s, r'; \frac{\omega}{c}\right)\right]. \tag{S22}$$

Here, r' is an arbitrary point inside Γ and $s \subset \Gamma$, and ∂_ν denotes the outward normal derivative. By pushing r' to be involved in Γ too, as $r' \to s' \subset \Gamma$, we can complete the BIE formulation. Note that we can circumvent the singular point of $G(s, s' = s)$ in terms of the Cauchy principal value. The constant multiplied to LHS is given as $\alpha \equiv \theta/2\pi$, in which $\theta = \pi$ for a convex-smooth boundary shape such as the one we are to deal with.

By meshing Γ into N number of segments, the BIE turns into a $2N \times 2N$ matrix equation of the BEM,

$$\left[\int_{\gamma_j} -G\left(s_i, s'_j; \frac{\omega}{c}\right)ds' \quad \left\{\int_{\gamma_j}\partial_\nu G\left(s_i, s'_j; \frac{\omega}{c}\right) - \frac{\delta_{ij}}{2}\right\}ds'\right]\left[\partial_\nu\psi(s'_j) \quad \psi(s'_j)\right]^T = 0, \tag{S23}$$

where $1 \leq i, j \in \mathbb{N} \leq N$, and γ_j is the segment length of the j-th element having a center position at $s'_j(x, y)$. Having fixed the boundary shape of Γ, which is divided by a proper number of element N, the matrix equation

$$M_{i,j}\,(\omega/c)\,\Psi = 0 \tag{S24}$$

produces, in principle, an infinite number of solutions for ω/c and corresponding vectors Ψ. The nonlinear eigenvalue problem Eq. (S24) cannot be solved directly through a one-step diagonalization for ω/c; instead, we have to iteratively find solutions E starting from a proper initial guess E_0. It is known that this root-search strategy functions well and is robust with the aid of several efficient numerical methods, e.g., those in (*9–12*), provided that E_0 is close enough to E. Nevertheless, preparing good quality initial guesses remains as a challenging task, and more critically, this approach can be found to be insufficient when it comes to computing the complete structure of photonic energy bands.

With this motivation, we adopt a more systematic approach that operates with the 'contour integral' of matrix M for $\omega/c \in \mathbb{C}$. Among several variations of this approach, we implement a block version of the Sakurai–Sugiura method (block SS) following (*13–17*). From a practical viewpoint, this method can provide numerous good quality initial guesses. The reason for using an additional refinement iterative process is because this method produces undesired solutions. Although it seems that the block SS method has yet to be well introduced in the optical BEM community, it is found in our work that this method is remarkably helpful to compute the eigenstates, particularly when we are to compute photonic band structures. We emphasize that the block SS method itself was originally designed to self-consistently give the exact solutions at once without extra iterative computations like we have done in our work. Below, forgoing the sophisticated mathematical proofs involved in this method, its essence is outlined.

S8 Block Sakurai–Sugiura contour integral method

Given the constructed matrix $M(\omega/c)$ for the BEM, we can define the moment matrix given as

$$\mu_j = \frac{1}{2\pi i}\oint_C z^j v^H M^{-1} v dz, \tag{S25}$$

where $z \leftarrow \omega/c \in \mathbb{C}$, $v \in \mathbb{C}^{2N\times k}$, $\{j,\ k\} \in \mathbb{N}$, and the contour C is set to enclose the target solutions of ω/c. Note that the entries in v are complex-valued random numbers. After obtaining μ_j, we can construct two Hankel matrices:

$$H_m^R = \begin{pmatrix} \mu_0 & \mu_1 & \cdots & \mu_{m-1} \\ \mu_1 & \mu_2 & \cdots & \mu_m \\ \vdots & \vdots & \ddots & \vdots \\ \mu_{m-1} & \mu_m & \cdots & \mu_{2m-2} \end{pmatrix}, \quad H_m^L = \begin{pmatrix} \mu_1 & \mu_2 & \cdots & \mu_m \\ \mu_2 & \mu_3 & \cdots & \mu_{m+1} \\ \vdots & \vdots & \ddots & \vdots \\ \mu_m & \mu_{m+1} & \cdots & \mu_{2m-1} \end{pmatrix}. \tag{S26}$$

According to the proofs in (*13*), the eigenvalues λ_l of the pencil

$$H_m^L = \lambda_l H_m^R\,,\ (l = 1, \ldots, m) \tag{S27}$$

are the same ones in our original BEM up to m, as

$$M_{i,j}(\lambda_l = \omega_l/c)\Psi = 0. \tag{S28}$$

Although we successfully transformed our original BEM into a one-step diagonalization problem, there are still some remaining challenges in this method. First, a good approximation of the contour integral in Eq. (S25); second, a proper choice of order j for the moment matrix μ_j; and third, a reliable column number k of vectors v. The first issue is concerned with the selection of the integral contour shape, with a numerical integration typically carried out through the conventional trapezoidal rule. In our work, we use an elliptical polygon path of the integral contour (*15, 16*), given as

$$\frac{\omega}{c} = \frac{\omega_0}{c} + \rho(\cos\theta_l + i\eta\sin\theta_l),\ \theta_l = \frac{2\pi}{\mathcal{N}}(l + 1/2), \tag{S29}$$

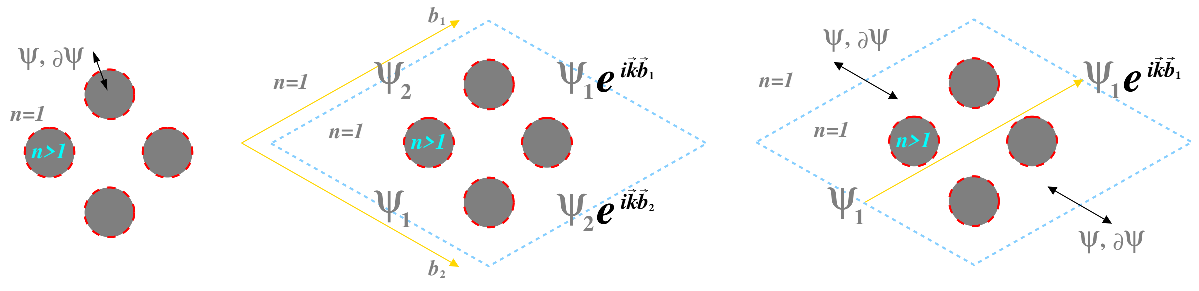

Figure S3: Schematic illustrations of the resonator-vacuum boundary (red dashed) and the vacuum-vacuum (blue dashed) virtual boundary that are used for computing band structures through the BEM. The latter boundary defines the primitive unit cell with the translation vectors $(\vec{b}_1, \vec{b}_2)$ for a two-dimensional periodic system and $\vec{b}_1$ for a one-dimensional one. In all boundary cases, the wave functions ψ and its normal derivative $\partial_\nu \psi$ are continuous. n and $\vec{k}$ represent the refractive index and the Bloch wave vector, respectively.

where $\mathcal{N}$ is the number of segments for the integral contour C. Then, the integral in Eq. (S25) is numerically calculated by a discrete sum (*15, 16*), as

$$\mu_j \approx \frac{1}{\mathcal{N}} \sum_{l=0}^{\mathcal{N}-1} \rho \left[\frac{\omega_l - \omega_0}{c\rho} \right]^l [\eta \cos\theta_l + i \sin\theta_l]\, v^H M^{-1} v. \tag{S30}$$

Since the scale factor η controls the width of the elliptical shape in the imaginary value direction, if the value is small, the contour C can tightly enclose the real axis in the complex plane, while the elliptical shape becomes circular when $\eta = 1$. As the contour C is shifted by ω_0/c and is scaled by ρ, our desired solution can be recovered after compensating this shift-scale over the obtained eigenvalue λ, as $\omega/c = \omega_0/c + \rho\lambda$.

S9 Periodic boundary conditions for 1D and 2D structures

Generally, the BEM is employed to compute eigenstates in isolated sub-systems embedded in an infinite-extended environment (see the leftmost schematic in Fig. S3). These eigenstates satisfy the Sommerfeld pure outgoing condition, which is adequate in the finite-size systems that we have examined in the main text to exemplify the edge and corner modes. To obtain the

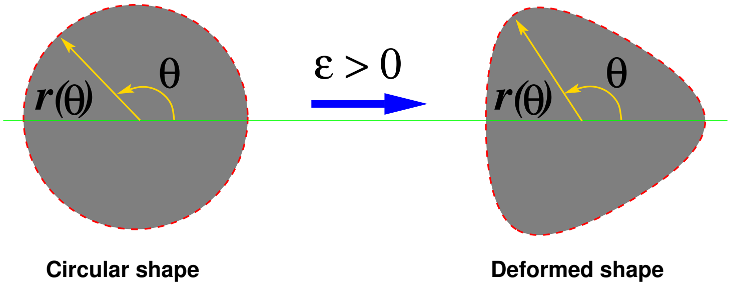

Figure S4: Deformed single resonator quasi-atom whose boundary is defined by Eq. (S31) with $(t, \theta_0) = (3, 0)$.

photonic energy band structures, we impose a Bloch boundary condition on the pairwise virtual boundaries (according to the translation vectors of the unit cell) constructed in the environmental domain outside the resonators. The composite domain of the primitive unit cell is defined by the actual system boundaries and these virtual boundaries. If the periodic boundary condition is set along the full two-dimensional translational vectors of the primitive unit cell (see the middle schematic in Fig. S3), we can obtain a series of energy dispersions as a function of the Bloch wavevectors.

A mixed boundary condition that combines the pure-outgoing situation at infinity and the periodicity is possible as well (see the rightmost schematic in Fig. S3). This kind of boundary condition is necessary for computing the band structure in a one-dimensional photonic crystal satisfying the open boundary condition along one of the primitive unit cell directions while also satisfying the periodic boundary condition along another translational vector direction.

S10 Boundary shape of a single resonator quasi-atom

The optical resonator quasi-atoms have two major system parameters: a refractive index inside the resonator and a boundary shape. The latter is controlled by a deformation parameter ε and is given as follows:

$$r(\theta;\varepsilon) = A(\varepsilon)\left[1 + \varepsilon\cos(t\theta + \theta_0)\right]\ ,\ \{\theta \in [0, 2\pi),\ t \in \mathbb{N}\}\,, \tag{S31}$$

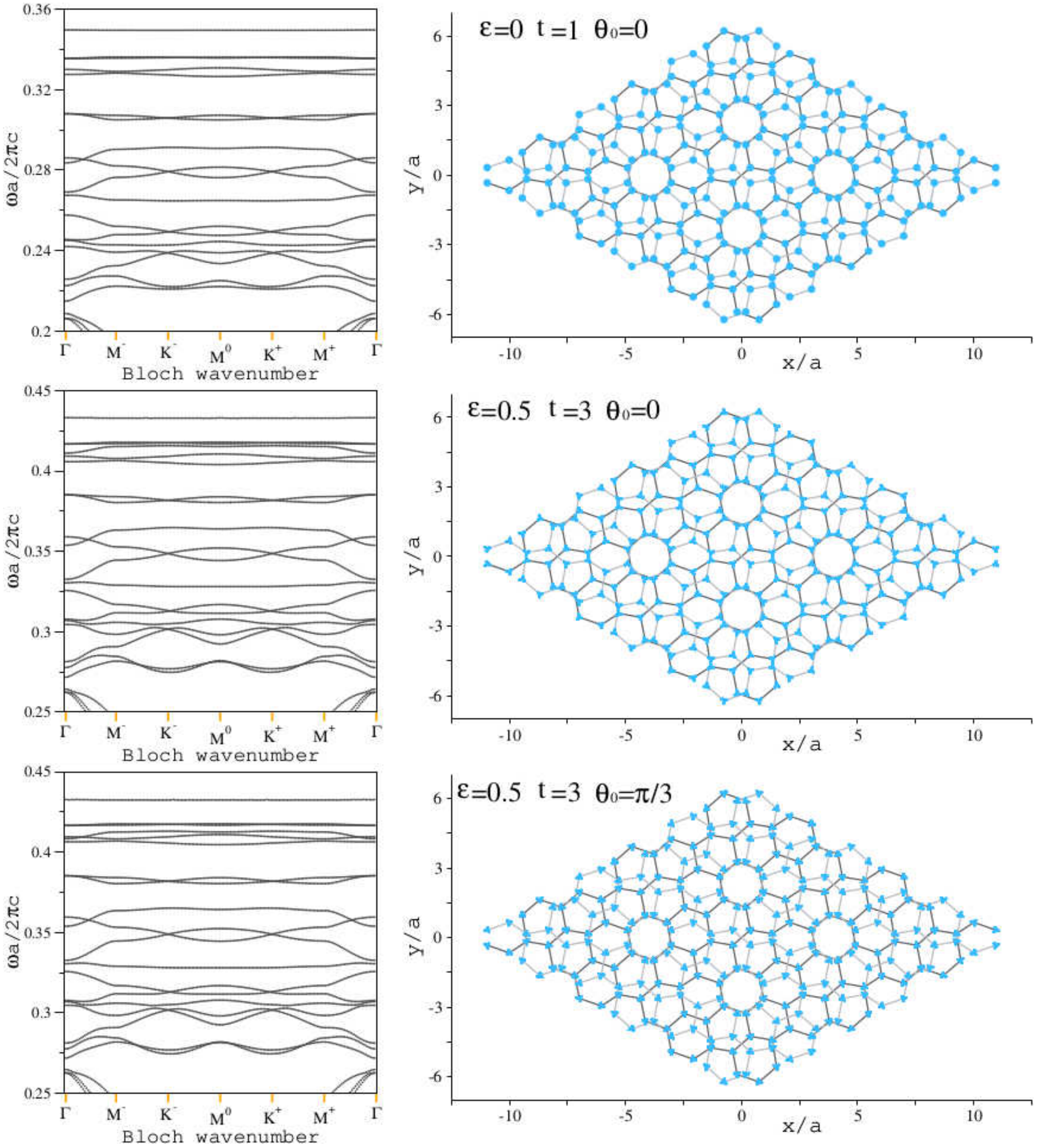

Figure S5: Energy bands (left) of a twisted-bilayer hexagonal moiré superlattice (right) consisting of circular (top) and C_3-symmetric deformed (middle and bottom) quasi-atoms. The refractive index of the resonator quasi-atoms is $n = 4$. The deformed shape of the quasi-atoms is given by Eq. (S31).

where $A(\varepsilon)$ stands for the normalization constant, t is the periodicity of the resonator shape (e.g., $t = 3$ for C_3 symmetry), and θ_0 the rotation of the resonator (see Fig. S4). It should be emphasized that although the results in our main text are discussed as if we focused only on the circular shape case (i.e., $\varepsilon = 0$), the overall band structures are robust against the deformation parameter ε (except for slight changes in the detailed structures caused by different resonator characteristics like area, perimeter length, curvature, etc.). More precisely, this robustness is observed in the Rayleigh scattering regime, in which the wavelength of the optical modes corresponding to low energy is larger than the resonator size ($\sim \sqrt{\text{resonator area}}$). In this regime, the deformation effects are suppressed and ultimately negligible since the resonator area is smaller than that of the first Mie resonance. As we can see in Fig. S5, the global structure of the bands is not sensitive to the deformation parameter. Note that all energy bands in Fig. S5 are computed with the refractive index $n = 4$ which is the same value used in the main text.

S11 Full characterization of the wave functions and parity eigenvalues at the TRIM points

Figures S6–S8 present the full wave characters at the TRIM points in the BZ. Examining the wavefunctions given below we can identify the mirror reflection symmetry $\{\mathcal{M}_x, \mathcal{M}_y\}$ and the inversion parity symmetry $\mathcal{M}_x \times \mathcal{M}_y = \mathcal{C}_{2z}$ that are the basis of the tables in Table S1,S2,S3,S4.

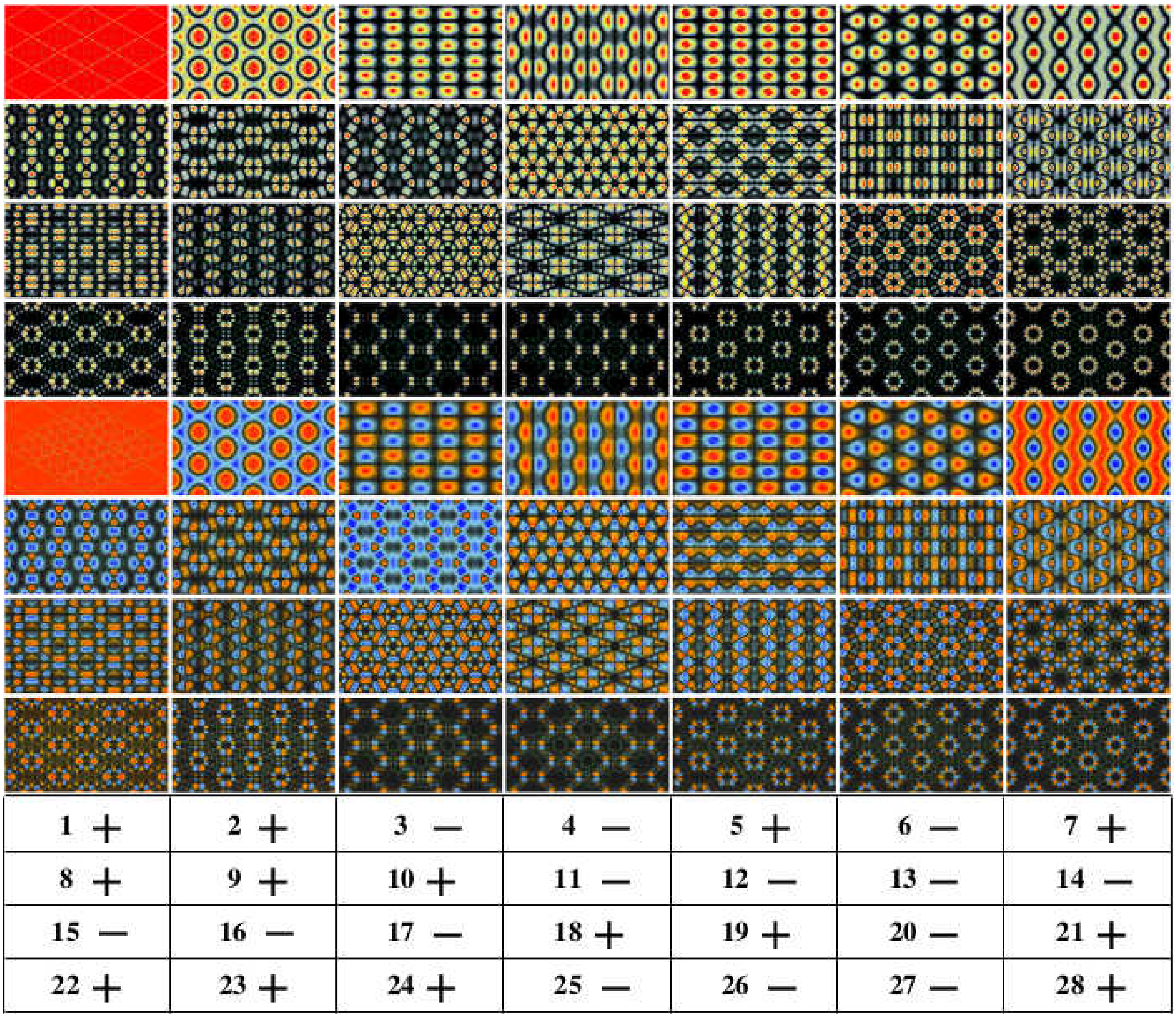

1 +	2 +	3 −	4 −	5 +	6 −	7 +
8 +	9 +	10 +	11 −	12 −	13 −	14 −
15 −	16 −	17 −	18 +	19 +	20 −	21 +
22 +	23 +	24 +	25 −	26 −	27 −	28 +

Figure S6: Upper 4 rows: Wave intensities $|\psi(\vec{r})|^2$ corresponding to the Bloch wavevector Γ and the energies ϵ_j for bands $j \in [1, 28]$. Next 4 rows: Real parts of the waves Re[$\psi(\vec{r}$] corresponding to the Bloch wavevector Γ and the energies ϵ_j for bands $j \in [1, 28]$. Last 4 rows: A table of the energy band number j and $\mathcal{C}_{2z}$ of the wavefunctions.

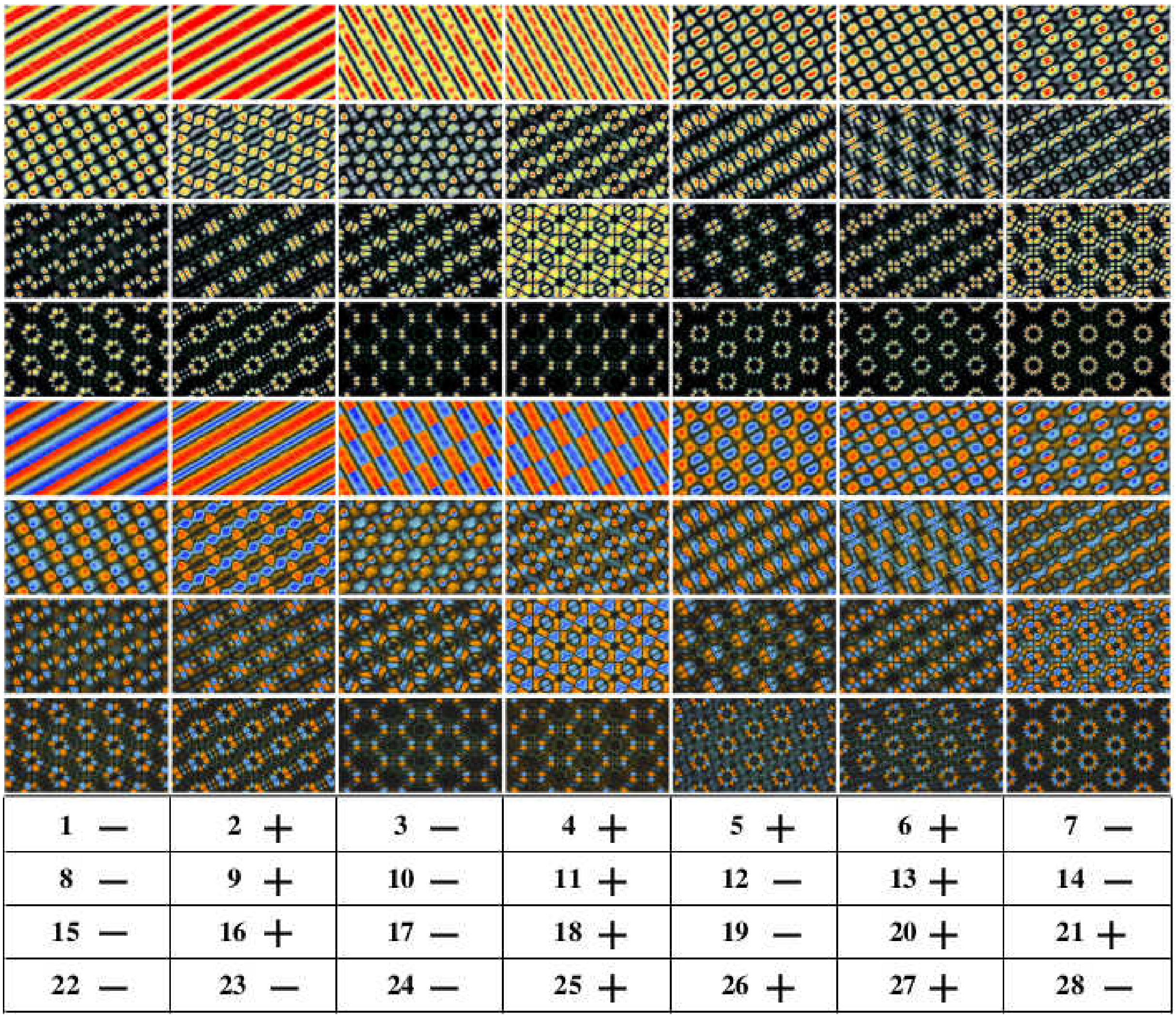

1 −	2 +	3 −	4 +	5 +	6 +	7 −
8 −	9 +	10 −	11 +	12 −	13 +	14 −
15 −	16 +	17 −	18 +	19 −	20 +	21 +
22 −	23 −	24 −	25 +	26 +	27 +	28 −

Figure S7: Upper 4 rows: Wave intensities $|\psi(\vec{r})|^2$ corresponding to the Bloch wavevector M^- and the energies ϵ_j for bands $j \in [1, 28]$. Next 4 rows: Real parts of the waves $\mathrm{Re}[\psi(\vec{r}]$ corresponding to the Bloch wavevector M^- and the energies ϵ_j for bands $j \in [1, 28]$. Last 4 rows: A table of the energy band number j and $\mathcal{C}_{2z}$ of the wavefunctions.

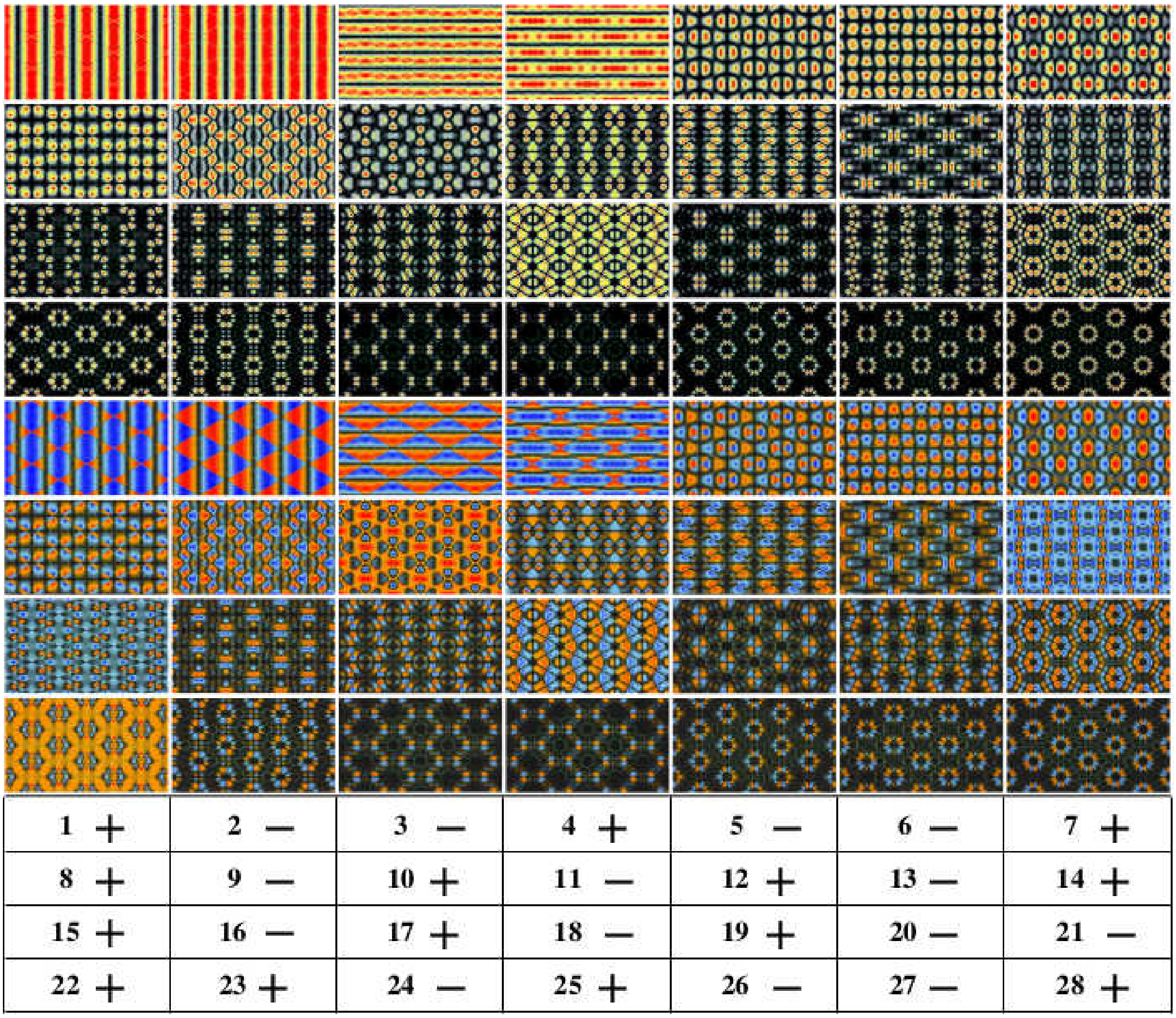

1 +	2 −	3 −	4 +	5 −	6 −	7 +
8 +	9 −	10 +	11 −	12 +	13 −	14 +
15 +	16 −	17 +	18 −	19 +	20 −	21 −
22 +	23 +	24 −	25 +	26 −	27 −	28 +

Figure S8: Upper 4 rows: Wave intensities $|\psi(\vec{r})|^2$ corresponding to the Bloch wavevector M^0 and the energies ϵ_j for bands $j \in [1, 28]$. Next 4 rows: Real parts of the waves $\mathrm{Re}[\psi(\vec{r}]$ corresponding to the Bloch wavevector M^0 Γ and the energies ϵ_j for bands $j \in [1, 28]$. Last 4 rows: A table of the energy band number j and $\mathcal{C}_{2z}$ of the wavefunctions.

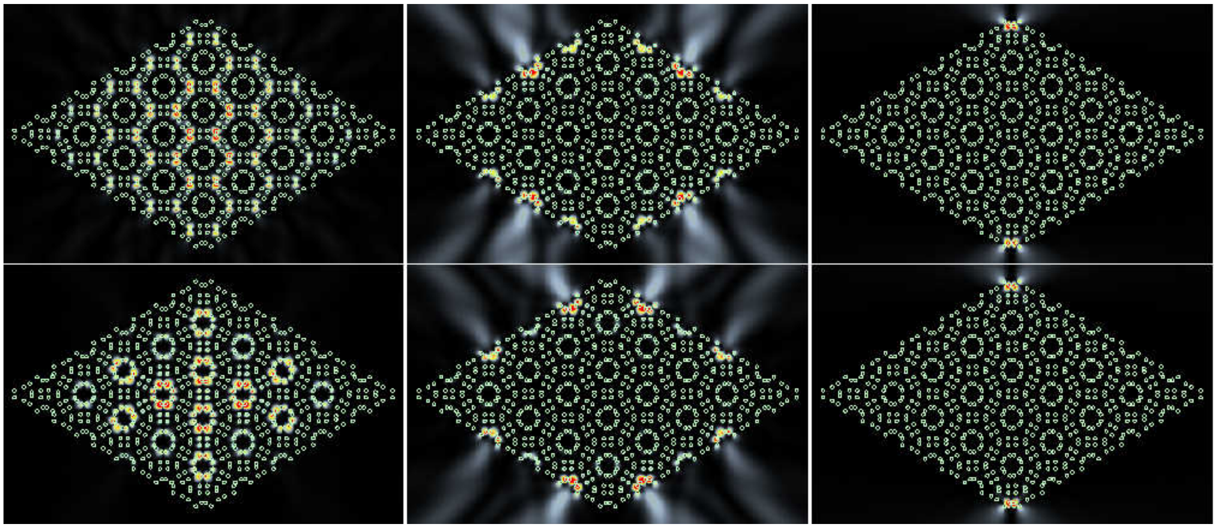

Figure S9: Wave intensities $|\psi|^2$ of different class eigenstates in a finite size "dielectric" system. The two rows of the left, middle, and right columns show the bulk, edge, and corner modes, respectively. The system consists of 5×5 unit cells of moire honycomb superlattices with a twist angle $21.78°$. The resonator quasi-atoms are made of the dielectric material with a refractive index $n = 4$.

S12 Bulk, edge, and corner modes in a finite size system under a "dielectric" boundary condition

For a clear demonstration of distinct wave confinement properties among different classes of the bulk, edge, and corner modes in a finite size "dielectric" system, we exemplify wave intensities of such different classes of modes in Fig. S9. In the edge and corner modes, it is emphasized that particular interesting features of the wave tails are observed. This observation clearly verifies that the extended waves into the homogeneous region in Fig. 3**c** in the main text are not numerical artifacts. The modes shown here are to further support our argument on the higher-order topological states associated with Fig. 4 in the main text.

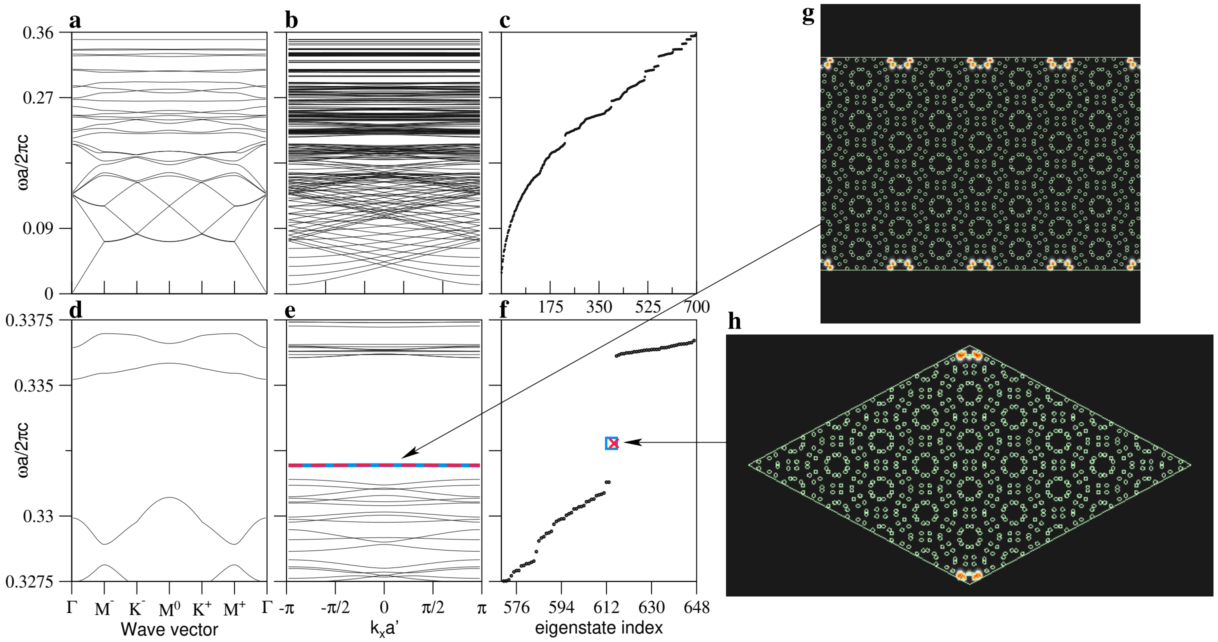

Figure S10: Energy bands of a 2D-periodic moire superlattice **a** and a 1D moire superlattice having a 5-cell width in the y-direction **b**. The 1D bands are obtained by applying the periodic boundary condition in the x-direction and the "Dirichlet" boundary condition on the upper and the lower bound of the system in the y-direction. **c** Energy eigenvalues in the finite size system satisfying the "Dirichlet" boundary condition on the outermost boundaries of systems. **d**, **e**, and **f** are the zooming of **a**, **b**, and **c**, respectively. **g** The edge mode in the 1D system, corresponding to the energy band indicated by the arrowed line. On the straight lines of the upper and lower boundaries, the "Dirichlet" boundary condition is applied. **h** Corner mode in the finite size system satisfying the "Dirichlet" boundary condition on the outermost boundaries of the system shown by the four straight edge lines. It corresponds to the energy eigenvalue marked by the arrowed line. The refractive index used is fixed as $n = 4$ for all cases.

S13 Edge and corner modes under the "Dirichlet" boundary condition

This section proves the robustness of the edge and corner modes independent of the boundary condition (B.C.). Since, the modes shown in Fig. 3 and Fig. 4 in the main text satisfy the "Dielectric" B.C. (i.e., open B.C.), the extended wave tails in the vacuum region is natural. By obtaining the corresponding edge and corner modes imposing the "Dirichlet" B.C. (i.e., closed B.C.) in Fig. S10, we demonstrate their consistent existence regardless of the open or closed

B.C.. Because the Dirichlet B.C. implies the vanishing waves outside the system, no waves can be observed in that region, as we can see in Figs. S10**g** and **h** (i.e., the region outside of the outermost straight lines). This invulnerable persisting property of our edge and corner modes is readily understood if we recall the fact that the modes have a topological origin. Note that the Dirichlet B.C. is sometimes called the perfect-electric-conductor (PEC) B.C. equivalently.

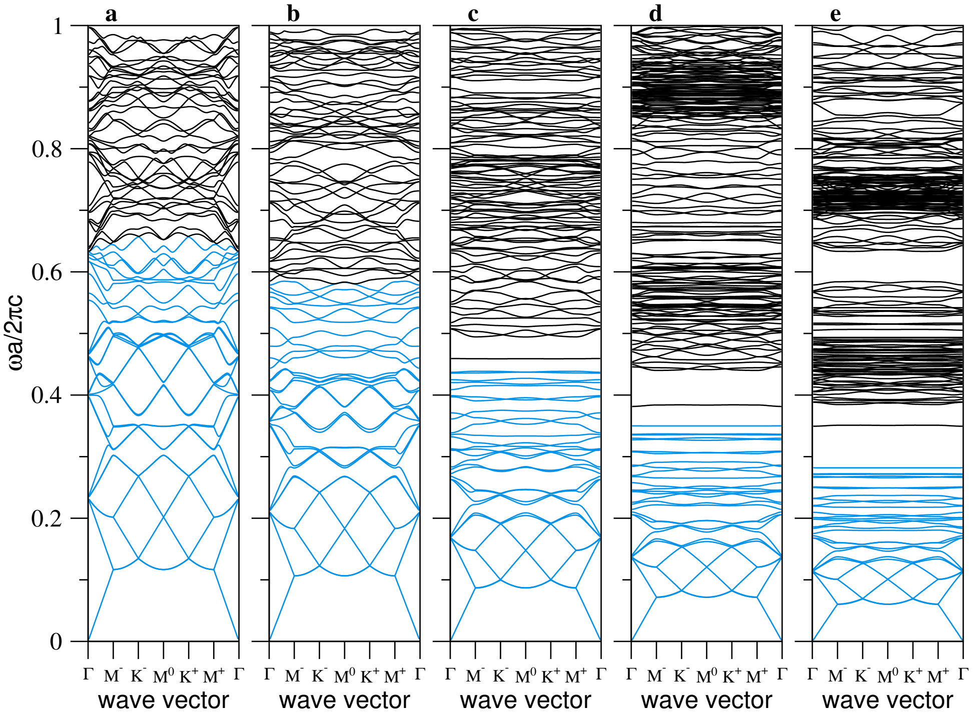

Figure S11: Refractive index-dependent energy bands of honeycomb moire superlattices with a twist angle $\theta = 21.78$. This twist angle correspond to the superlattice containing 28 atomic sites in a single unit cell. The bands are obtained for different resonator refractive indexes **a** $n = 1.5$; **b** $n = 2$; **c** $n = 3$; **d** $n = 4$; and **e** $n = 5$. The lowest 28 bands are marked by the blue color.

S14 Energy bands depending on the refractive index of resonator quasi-atoms

In this section, the refractive index-dependent energy bands are examined to show that we can classify the lowest 28 bands into a single group, distinguished from other higher energy bands, when the refractive index increases. Figure S11 demonstrates that the lowest 28 energy bands (blue curves) are distinguishable from the above ones (black curves) in the higher n case (i.e., $n \geq 3$). On the other hand, those in the lower n case (i.e., $n \leq 2$) are indistinguishable as they are mixed severely. We can attribute this observation to the enhanced confinement of modes inside the resonator when its refractive index has higher values.

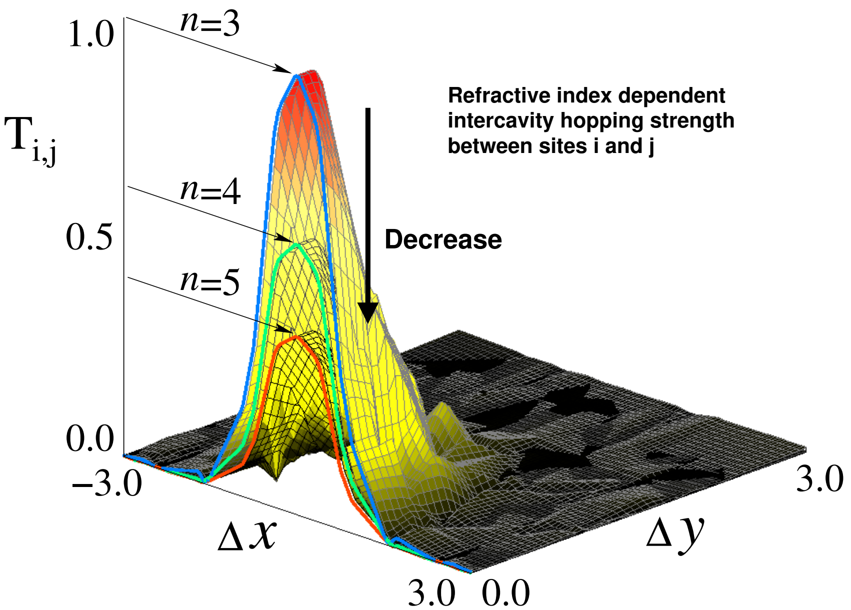

Figure S12: Refractive index-dependent effective hopping strength $\mathrm{T}_{i,j}$ as a function of the displacement, $(\Delta x, \Delta y) = (x_i - x_j, y_i - y_j)$, between two quasi-atoms (i, j). $\mathrm{T}_{i,j}$ is computed by means of the same procedures used in obtaining Fig. S1 **b**.

S15 Hopping strength depending on the refractive index

To reveal the refractive index-dependent hopping strength, in this section, the hopping strength $\mathrm{T}_{i,j}$ is computed in Fig. S12 based on the bands in Fig. S10, together with the wave functions corresponding to those bands. As the figure shows, the hopping strength is significantly suppressed when the refractive index increases. This result is consistent with the band separation results in the higher refractive index regime, which are shown in Fig. S11. Therefore, they further support our arguments associated with Fig.2 **b** in the main text, proving the validity of our claim in the broader range of the refractive index. Figure S12 verifies that (i) the inter-atomic coupling profiles are qualitatively identical [i.e., the envelope shape of $T(dx, dy)$] in the higher refractive index regime $(n > 3)$, and (ii) the modulus of the coupling strength is, roughly speaking, inverse proportional to the refractive index, i.e., $\sim (1/n)$.

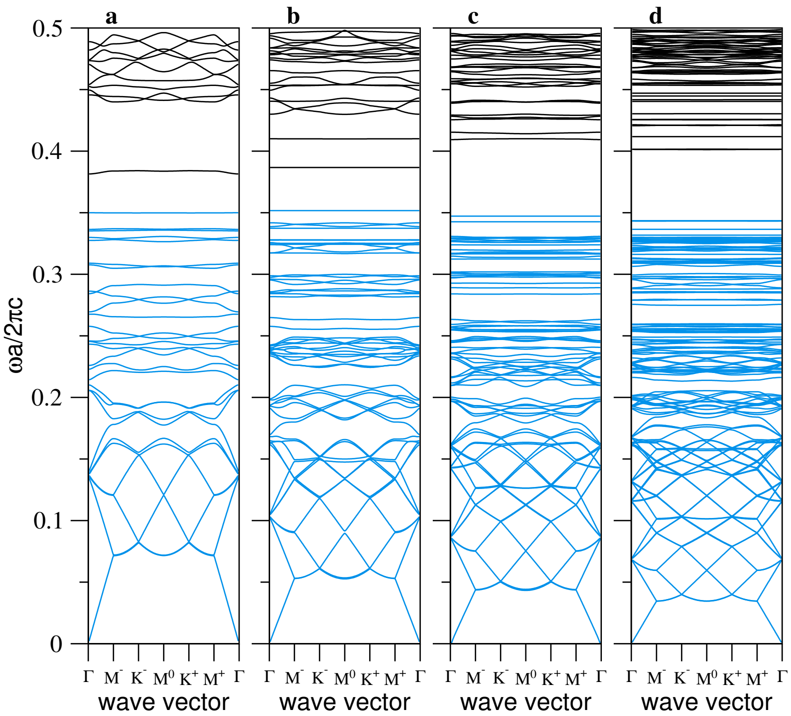

Figure S13: Twist angle-dependent energy bands of honeycomb moiré superlattices with a twist angle **a** $\theta = 21.78°$; **b** $\theta = 27.79°$; **c** $\theta = 13.17°$; and **d** $\theta = 17.89°$, containing, respectively, 28; 52; 76; and 124 atomic sites in a single unit cell. The lowest 28, 52, 76, and 124 bands are marked by the blue color. The refractive index is fixed as $n = 4$.

S16 Twist angle-dependent energy bands

To demonstrate the parametric consistency of our arguments about the separation of the lowest band group and the flat bands concerning the different twist angles of moire honeycomb superlattices, the twist angle-dependent energy bands are examined in Fig. S13, for a fixed refractive index $n = 4$. The different numbers of the lowest band groups (blue curves) in the figure imply that the changing of twist angle induces nonidentical size moiré unit cells containing different numbers of atomic sites in a single unit cell. Despite this different unit cell size, it is consistently clear that the set of the lowest band groups are well-separated from the higher energy bands (black curves). In addition, a series of flat bands are also evident in the higher energy regime among the blue curves.

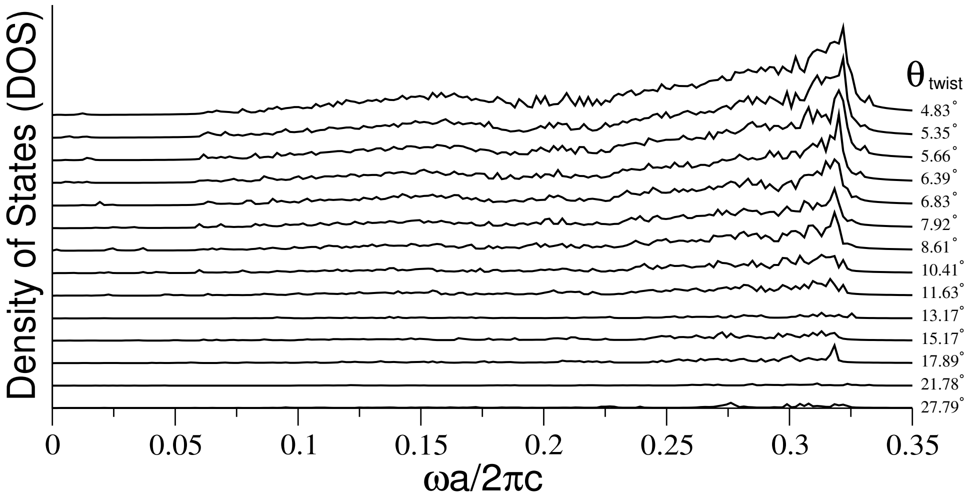

Figure S14: Twist angle-dependent density of states: $\mathrm{DOS}(\Omega) \equiv \sum_{\Omega_0}(\Omega - \Omega_0 + i\gamma)^{-1}$ with a constant width $\gamma = 5 \times 10^{-5}$, where $\Omega \equiv \omega a/2\pi c$. DOSs are obtained from the twist angles-dependent band structures, which are constructed based on the extension of the coupling strength shown in Fig. 2**b** in the main text and Fig. S12 in this Supplementary Information. The refractive index is set as $n = 4$ for all cases.

S17 Twist angle-dependent density of states (DOS)

In this section, the twist angle-dependent density of states (DOS) is exemplified for the moiré honeycomb superlattices with a fixed refractive index $n = 4$, as shown in Fig. S14. By means of the tight-binding models, the twist angles {21.79°, 21.78°, 17.89°, 15.17°, 13.17°, 11.63°, 10.41°, 8.61°, 7.92°, 6.83°, 6.39°, 5.66°5.35°, 4.83°} are investigated from the lower to higher curves in the figure. For the numerical computations, the basic couplings of the displacement-dependent hopping strength are deduced from the ones obtained in Fig. S12 in this Supplementary Information and in Fig. 2**b** in the main text. In Fig. S14, the clear separations between the lowest band group and the higher energy bands (by abrupt vanishing DOS at around $\omega a/2\pi c \sim 0.325$), as well as a series of flat bands (imprinted in a number of intense sharp peaks) for various twist angles are revealed.

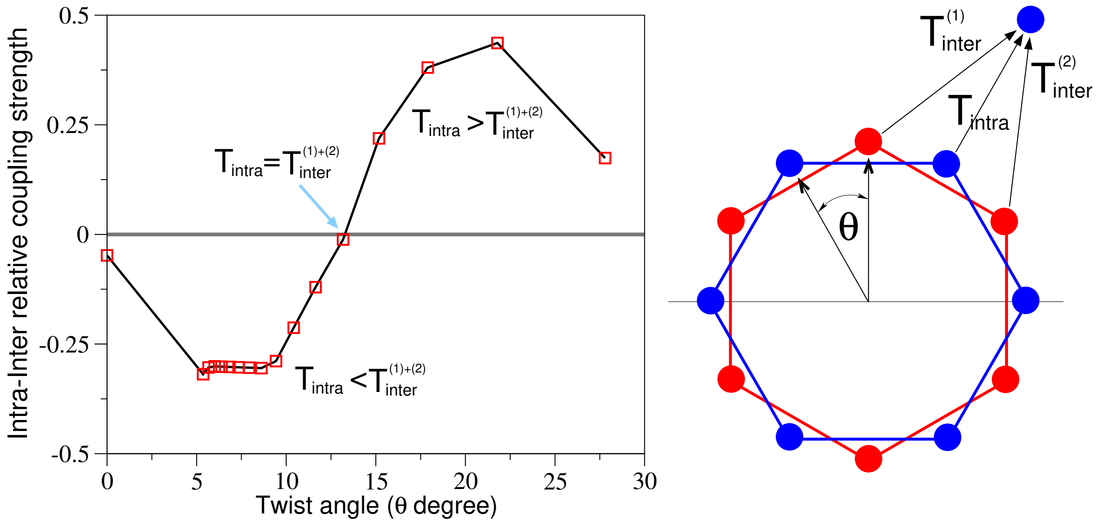

Figure S15: Twist angle-dependent competing coupling strength given as a scaled differences between the intralayer and interlayer couplings: $y|_\theta \equiv \left[T_{\text{intra}} - T_{\text{inter}}^{(1)+(2)}\right] / T_{\text{intra}}$, where $T_{\text{inter}}^{(1)+(2)} \equiv T_{\text{inter}}^{(1)} + T_{\text{inter}}^{(2)}$. For a transparent verification of the twist angle-resolved tunability of the coupling strength balance between the intra and interlayer couplings, here, we simplify the coupling configurations focusing only on the innermost dodecagonal atomic sites and the outer target sites.

S18 Tunable intra and interlayer couplings as a function of the twist angle

This section explicitly demonstrates a competing property of the intra and interlayer couplings depending on the twist angle of the moiré honeycomb superlattice. Figure S15 shows that we can fine-tune the intralayer and interlayer coupling strength by changing the twist angle to achieve perfect destructive interference between the two couplings. As the intralayer and interlayer couplings are identical around $\theta \sim 15°$, the optimal condition for the Aharonov-Bohm cage effect corresponding to the perfect flat bands is fulfilled. The refractive index used in the figure is $n = 4$.

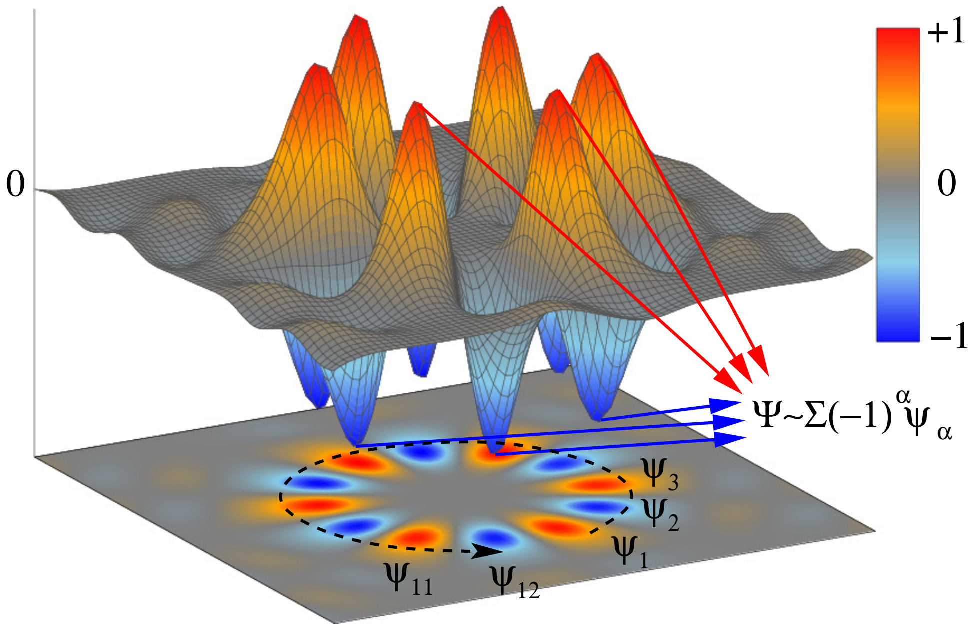

Figure S16: Wave function, Re(ψ), of the innermost dodecagonal 12 quasi-atoms forming a localized standing wave. The summed contributions of the alternating phase waves ψ_α [red arrows from (+) sign; blue arrows from (-) sign] result in the destructive interference outside the innermost dodecagonal quasi-atoms, where α is a site index $(1, 2, 3, \cdots, 12)$.

S19 Strongly localized waves on the innermost dodecagonal quasi-atoms associated with flat bands

For more lucid substantiation of Eq (3), shown in the main text, we visualize the anomalously localized states of alternating phase waves on the innermost dodecagonal quasi-atoms near the rotation center. This representation is consulted to account for our argument on the localized state, shown in Fig. 3**a** in the main text, in terms of the Aharonov-Bohm cage effect. As the alternating phase of the standing waves gives rise to the destructive interference outside the dodecagonal quasi-atoms region, this disjoint character of the state brings about the emergence of flat bands. In other words, the standing waves cannot propagate further to the outer atomic sites, as the sign change in the wave functions produces destructive interference to the outer sites (red and green arrows in Fig. 3**b** in the main text). The concrete localization property

of this state is clearly demonstrated in Fig. 3**a** in the main text, as well as by 28th waves in Figs. S6, S7, and S8 in this Supplementary Information. Furthermore, as are well-visible in Fig. 2**a** in the main text and Fig. S5 in this Supplementary Information, the bands corresponding to the 28th state (i.e., the highest energy band in the figures) manifest the almost flat bands (the Bloch vector-dependent dispersions of the bands are negligible and hard to be resolved by naked eyes).

References

1. A. P. Schnyder, S. Ryu, A. Furusaki, A. W. Ludwig, Classification of topological insulators and superconductors in three spatial dimensions, *Phys. Rev. B* **78**, 195125 (2008).
2. J. C. Y. Teo, C. L. Kane, Topological defects and gapless modes in insulators and superconductors, *Phys. Rev. B* **82**, 115120 (2010).
3. B. Bradlyn, *et al.*, Topological quantum chemistry, *Nature* **547**, 298 (2017).
4. M. G. Vergniory, *et al.*, Graph theory data for topological quantum chemistry, *Phys. Rev. E* **96**, 023310 (2017).
5. L. Elcoro, *et al.*, Double crystallographic groups and their representations on the Bilbao Crystallographic Server, *Journal of Applied Crystallography* **50**, 1457 (2017).
6. J. Wiersig, Boundary element method for resonances in dielectric microcavities, *J. Opt. A: Pure Appl. Opt.* **5**, 53 (2002).
7. P. A. Knipp, T. L. Reinecke, Boundary-element method for the calculation of electronic states in semiconductor nanostructures, *Phys. Rev. B* **54**, 1880 (1996).
8. J. R. de Lasson, *et al.*, Benchmarking five numerical simulation techniques for computing resonance wavelengths and quality factors in photonic crystal membrane line defect cavities, *Opt. Express* **26**, 11366 (2018).
9. G. Veble, T. Prosen, M. Robnik, Expanded boundary integral method and chaotic time-reversal doublets in quantum billiards, *New J. Phys.* **9**, 15 (2007).
10. R. Dubertrand, E. Bogomolny, N. Djellali, M. Lebental, C. Schmit, Circular dielectric cavity and its deformations, *Phys. Rev. A* **77**, 013804 (2008).

11. J. Kullig, J. Wiersig, Perturbation theory for asymmetric deformed microdisk cavities, *Phys. Rev. A* **94**, 043850 (2016).

12. C.-L. Zou, H. G. L. Schwefel, F.-W. Sun, Z.-F. Han, G.-C. Guo, Quick root searching method for resonances of dielectric optical microcavities with the boundary element method, *Opt. Express* **19**, 15669 (2011).

13. J. Asakura, T. Sakurai, H. Tadano, T. Ikegami, K. Kimura, A numerical method for nonlinear eigenvalue problems using contour integrals, *JSIAM Letters* **1**, 52 (2009).

14. H. Isakari, T. Takahashi, T. Matsumoto, Periodic band structure calculation by the sakurai–sugiura method with a fast direct solver for the boundary element method with the fast multipole representation, *Eng. Anal. Bound. Elem.* **68**, 42 (2016).

15. C.-J. Zheng, H.-F. Gao, L. Du, H.-B. Chen, C. Zhang, An accurate and efficient acoustic eigensolver based on a fast multipole bem and a contour integral method, *J. Comput. Phys.* **305**, 677 (2016).

16. T. Sakurai, Y. Futamura, H. Tadano, Efficient parameter estimation and implementation of a contour integral-based eigensolver, *J. Algorithms Comput. Technol.* **7**, 249 (2013).

17. H. Gao, T. Matsumoto, T. Takahashi, H. Isakari, Eigenvalue analysis for acoustic problem in 3d by boundary element method with the block sakurai–sugiura method, *Eng. Anal. Bound. Elem.* **37**, 914 (2013).
